# Amelioration of Chromium-Induced Oxidative Stress by Combined Treatment of Selected Plant-Growth-Promoting Rhizobacteria and Earthworms *via* Modulating the Expression of Genes Related to Reactive Oxygen Species Metabolism in *Brassica juncea*

**DOI:** 10.3389/fmicb.2022.802512

**Published:** 2022-04-06

**Authors:** Pooja Sharma, Rekha Chouhan, Palak Bakshi, Sumit G. Gandhi, Rupinder Kaur, Ashutosh Sharma, Renu Bhardwaj

**Affiliations:** ^1^Department of Microbiology, DAV University, Jalandhar, India; ^2^Department of Botanical and Environmental Sciences, Guru Nanak Dev University, Amritsar, India; ^3^Indian Institute of Integrative Medicine (CSIR), Jammu, India; ^4^Department of Biotechnology, DAV College, Amritsar, India; ^5^Faculty of Agricultural Sciences, DAV University, Jalandhar, India

**Keywords:** *Burkholderia gladioli*, *Eisenia fetida*, reactive oxygen species, *Pseudomonas aeruginosa*, oxidative stress, glutathione, ascorbate, antioxidant enzyme

## Abstract

Chromium (Cr) toxicity leads to the enhanced production of reactive oxygen species (ROS), which are extremely toxic to the plant and must be minimized to protect the plant from oxidative stress. The potential of plant-growth-promoting rhizobacteria (PGPR) and earthworms in plant growth and development has been extensively studied. The present study was aimed at investigating the effect of two PGPR (*Pseudomonas aeruginosa* and *Burkholderia gladioli*) along with earthworms (*Eisenia fetida*) on the antioxidant defense system in *Brassica juncea* seedlings under Cr stress. The Cr toxicity reduced the fresh and dry weights of seedlings, enhanced the levels of superoxide anion (O_2_•^–^), hydrogen peroxide (H_2_O_2_), malondialdehyde (MDA), and electrolyte leakage (EL), which lead to membrane as well as the nuclear damage and reduced cellular viability in *B. juncea* seedlings. The activities of the antioxidant enzymes, *viz*., superoxide dismutase (SOD), guaiacol peroxidase (POD), ascorbate peroxidase (APOX), glutathione peroxidase (GPOX), dehydroascorbate reductase (DHAR), and glutathione reductase (GR) were increased; however, a reduction was observed in the activity of catalase (CAT) in the seedlings under Cr stress. Inoculation of the PGPR and the addition of earthworms enhanced the activities of all other antioxidant enzymes except GPOX, in which a reduction of the activity was observed. For total lipid- and water-soluble antioxidants and the non-enzymatic antioxidants, *viz*., ascorbic acid and glutathione, an enhance accumulation was observed upon the inoculation with PGPR and earthworms. The supplementation of PGPR with earthworms (combined treatment) reduced both the reactive oxygen species (ROS) and the MDA content by modulating the defense system of the plant. The histochemical studies also corroborated that the combined application of PGPR and earthworms reduced O_2_•^–^, H_2_O_2_, lipid peroxidation, and membrane and nuclear damage and improved cell viability. The expression of key antioxidant enzyme genes, *viz*., *SOD, CAT, POD, APOX, GR, DHAR*, and *GST* showed the upregulation of these genes at post-transcriptional level upon the combined treatment of the PGPR and earthworms, thereby corresponding to the improved plant biomass. However, a reduced expression of *RBOH1* gene was noticed in seedlings supplemented under the effect of PGPR and earthworms grown under Cr stress. The results provided sufficient evidence regarding the role of PGPR and earthworms in the amelioration of Cr-induced oxidative stress in *B. juncea*.

## Introduction

In the recent past, rapid industrialization and increased urbanization led to an enhanced level of heavy metal contamination in the environment, which has emerged as a global concern (Saleem et al., 2018). Soil contamination with heavy metals is a major concern due to their non-biodegradable nature, bio-accumulation, and persistence in the environment (Din et al., 2020). Chromium (Cr) contamination has increased in the soil and into the water because of the release of Cr containing effluents from various industries such as electroplating, alloying, metallurgy, tannery, textile dyes, paints, and timber processing (Ganesh et al., 2009; Gill et al., 2016). Cr exists in six oxidation states, but Cr (III) and Cr (VI) are the most stable forms in the aquatic and terrestrial environments (Santos et al., 2009; Augustynowicz et al., 2010). Cr (VI) is considered extremely toxic, as it is a strong oxidizing agent having high redox potential, which is responsible for the prompt production of reactive oxygen species (ROS) (Shanker et al., 2005). Due to its highly soluble nature and capacity to pass through the plasma membrane, it enters the cytoplasm and reacts with intracellular structures (Ma et al., 2016). Increased deposition of Cr in soil results in its accumulation in plants, which affects various physiological and biochemical activities (Gangwar et al., 2011; Wang et al., 2013; Askari et al., 2021). Cr is a biologically non-essential element and shows toxicity beyond certain limits. Soil is known to accumulate as high as 2.6% (25,900 mg/kg) chromium (Palmer and Wittbrodt, 1991). The Cr plants exposed to Cr contamination have a marked reduction in growth and biomass (Qureshi et al., 2020). It is not required for the plant metabolism and impairs the growth and development of the plant at the cellular, organ, and at genetic level (Dixit et al., 2002; Wakeel et al., 2018). Cr exposure affects the plant metabolism, seed germination, photosynthesis, nutrient balance, water status, and antioxidant enzymes (Tripathi et al., 2015; Mahmud et al., 2017). Cr phytotoxicity is attributed to the enhanced production of ROS comprising superoxide anion (O_2_•^–^), hydrogen peroxide (H_2_O_2_), hydroxyl radical (OH•), and singlet oxygen (O_1_) species as a remedial strategy adapted by plants to alter the cellular redox status of plant, which results in severe oxidative damage (Ali et al., 2015; Ma et al., 2017). Since ROS are quite reactive, therefore, they may oxidize the cellular macromolecules like lipids, proteins, nucleic acids leading to oxidative burst, causing cellular damage, electrolyte leakage, and cell death (Apel and Hirt, 2004; Huang et al., 2014; Demidchik, 2015; Sharma et al., 2019; Gupta et al., 2020). Plasma-membrane-bound reduced nicotinamide adenine dinucleotide phosphate (NADPH) oxidase, also called as respiratory burst oxidase homolog (RBOH), is one the major enzymes responsible for the ROS generation under plant stress (Sharma et al., 2021). Accretion of malondialdehyde (MDA) level, a product of lipid peroxidation, is a potential indicator of oxidative damage under Cr stress (Anjum et al., 2017; Sallah-Ud-Din et al., 2017; Habiba et al., 2019).

Plants have complex and coordinated antioxidant defense system to maintain a steady-state level of ROS by scavenging the ROS generated to cope up the oxidative stress (Gill and Tuteja, 2010; Ma et al., 2017; Ashraf et al., 2021). The defense system comprising enzymes such as superoxide dismutase (SOD), catalase (CAT), ascorbate peroxidase (APOX), guaicol peroxidase (POD), dehydroascorbate reductase (DHAR), monodehydroascorbate reductase (MDHAR), glutathione reductase (GR), and glutathione peroxidase (GPOX) plays an imperative role in scavenging ROS produced due to metal toxicity (Gill and Tuteja, 2010). Numerous studies have reported altered activities of various antioxidant enzymes in different plant species under Cr stress (Gill et al., 2015; Kanwar et al., 2015; Handa et al., 2018). Non-enzymatic antioxidants (ascorbic acid, tocopherols, glutathione) also work in a coordinated manner with enzymatic antioxidants to neutralize Cr-induced ROS. For instance, the level of glutathione and ascorbic acid are increased under Cr stress (Ashger et al., 2018; Ulhassan et al., 2019). These antioxidants minimize the ROS- induced oxidative damage by acting as quenchers of ROS and lipid radicals (Noctor and Foyer, 1998; Smirnoff, 2000; Holländer-Czytko et al., 2005). *Brassica juncea* L. is an oil-yielding crop having medicinal properties. It is fast growing, produces a large amount of biomass, and possesses a sturdy and well-studied antioxidant defense system. Heavy metal stress results in a significant loss of its yield (Bhuiyan et al., 2011; Shekhawat et al., 2012).

Plant-growth-promoting rhizobacteria (PGPR) colonizing the rhizosphere of the plant have auspicious role in promoting plant growth and endurance and alleviating toxicity or injury to plants under heavy metal stress (Etesami and Maheshwari, 2018). The rhizospheric zone of the growing plants, rich in nutrients due to accretion of plant exudates, is a hotspot for microbial activity in the soil (Gray and Smith, 2005; De la Fuente Cantó et al., 2020). Plant-associated microorganisms such as rhizobacteria secrete various metabolites in the vicinity of plant roots that endorse the plant growth and development under stressed conditions (Kumar and Verma, 2018; Khoshru et al., 2020). These beneficial bacteria have the potential to mitigate the heavy metal toxicity by improved plant growth, reduced oxidative stress through altered activities of enzymatic antioxidants, and enhanced synthesis of non-enzymatic antioxidants (Islam et al., 2014). Implication of PGPR in improving plant health and alleviating heavy metal toxicity in plants unveils their potential for safe food production (Etesami and Beattie, 2017). Recently, augmentation of *Bacillus cereus* has been found to lower the MDA level and maintain membrane integrity in *Brassica nigra* under Cr stress (Akhtar et al., 2021). Supplementation of PGPR enhanced the tolerance of tomato plant to oxidative damage from Cr stress by the enhanced levels of enzymatic and non-enzymatic antioxidants (Gupta et al., 2020).

Soil microorganisms (like PGPR) have a positive interaction with macroorganisms (like earthworms), which plays an important role in organic matter decomposition and nutrient cycling (Brown et al., 2000; Lavelle, 2002). Earthworms have a positive impact on the growth of the plants (Kaur et al., 2017; Mahohi and Raiesi, 2021), which is owed to signal molecules released in the soil possibly due to the activation of PGPR (Elmer, 2009; Puga-Freitas et al., 2012). Humic acid and vermi-wash secreted by the earthworms are rich in nutrients, hormones, vitamins, and enzymes, which promote plant growth and development (Varghese and Prabha, 2014; Sharma et al., 2020). Plant growth is improved indirectly by the earthworms through the stimulation of the defense system of the plant (Blouin et al., 2005; Kaur et al., 2019).

PGPR and earthworms naturally coinhabit the soil and have an imperative role in nutrient acquisition and improving plant growth (Abd El and Bashandy, 2019; Mahohi and Raiesi, 2019). Numerous authors have reported the potential of combined inoculation of earthworms and PGPR in plant growth and development and nutrient acquisition (Edith Castellanos Wu et al., 2012; Suarez et al., 2014; Abd El and Bashandy, 2019). Although it is evident from the existing literature that the PGPR alleviates the harmful effects of heavy metals by strengthening the antioxidant defense system and endorsing plant growth; however, to the best of our knowledge, there is no information available on the potential of the combined treatment by PGPR and earthworms in ameliorating heavy metal stress, especially Cr. Therefore, the present study was conducted to investigate the role of PGPR (*Pseudomonas aeruginosa*, *Burkholderia gladioli*) and earthworms (*Eisenia fetida*), alone and in combination, on the mitigation of Cr stress in *Brassica juncea* to study their effect on plant biomass, stress-related biochemical changes, ROS generation, and the modulation of the antioxidant defense system. The results of biochemical assays are further supported by the histochemical staining methods for visualization of stress bio-markers *in situ*. Therefore, to the best of our knowledge, it is the first attempt to study the Cr stress ameliorative potential of the combined treatment of PGPR and Earthworms using three different approaches, *viz*., changes in cellular stress biochemistry, *in situ* visualization of stress biomarkers, and the changes in the expression of genes involved in ROS metabolism, to get a holistic insight into the amelioration process.

## Materials and Methods

### Microbial Culture

*Pseudomonas aeruginosa* MTCC7195 and *B. gladioli* MTCC10242 (obtained from Microbial Type Culture Collection, IMTECH Chandigarh, India) were revived in Nutrient Broth Medium (HiMedia Laboratories, Mumbai, India). Bacterial strains were incubated at 28°C for 24–48 h. The bacterial suspension was centrifuged at 4,000 rpm for 10 min at 4°C to obtain the pellet. The pellet was washed twice and resuspended in sterile distilled water to get uniform density of 10^9^ cells/ml.

### Plant Material and Experimental Design

*Brassica juncea* L., variety RLC-3, were obtained from Punjab Agriculture University, Ludhiana. Seeds were surface sterilized using sodium hypochlorite (0.5% v/v) followed by repeated washing with sterilized DW. The experiments were conducted under controlled conditions in plastic pots. Soil was mixed with organic manure in the ratio of 2:1. The soil mixture was autoclaved at 121°C for 30 min. The autoclaved soil (300 g) was poured into each plastic pot and amended with 0.5 mM Chromium solution in the form of potassium chromate (K_2_CrO_4_) obtained from Qualigens, Mumbai, India. Pots were inoculated with earthworms (three earthworms per pot) and bacterial suspension at the concentration of 109 cells/ml. The following experimental combinations were made: Control (Cn), Cr, CrE, CrM1, CrM2, CrM1M2, CrEM1, CrEM2, and CrEM1M2. Once the seeds were sown, the pots were maintained for 10 days in a seed germinator at 25 ± 2°C with a light intensity of 175 μmol m^–2^ s^–1^ and photoperiod of 16 h. The seedlings were harvested after 10 days, washed thoroughly with distilled water for various the morphological, biochemical, histochemical, and gene expression analysis. All the analysis for each treatment was conducted in triplicates.

### Determination of Bio-Mass

Bio-mass was measured in terms of fresh and dry weight of 10-day-old *B. juncea* seedlings. Weight of freshly harvested seedlings was recorded and considered as fresh weight. Dry weight of seedlings was determined by drying them in an oven at 60°C for 48 h.

### Assessment of Electrolyte Leakage

Electrolyte leakage (EL) was measured by following the method of Ahmad et al. (2016). Seedlings were placed in test tubes comprising 15 ml deionized water. The test tubes were placed in a shaking incubator at 30°C for 4 h, and then, the electrical conductivity (EC1) of the initial medium was measured. Afterward, all the test tubes were placed in an autoclave at 121°C for 20 min followed by cooling up to 25°C, and electrical conductivity (EC2) was measured and calculated by using the following formula:


(1) $$\text{EL}=\frac{\mathrm{EC1}}{\mathrm{EC2}}\times 100$$

### Estimation of Oxidative Stress Markers

#### Superoxide Anion Content

Superoxide content (O_2_.-) was determined by the method of Wu et al. (2010). One gram of fresh seedlings was homogenized with 3 ml of phosphate buffer 65 mM, pH 7.8, and then centrifuged at 12,000 rpm for 10 min. A supernatant (0.5 ml) was collected, and 0.1 ml of 10 mM hydroxylamine hydrochloride was added, followed by incubation at 25°C for 30 min. Afterward, 1 ml of 3-aminobenzenesulphonic acid and 1 ml of 1-naphthylamine were added to the reaction mixture. This mixture was again incubated at 25°C for 20 min, and the absorbance was read at 530 nm. The concentration of superoxide anion was determined using sodium nitrite as a standard and expressed as μmol g^–1^ FW.

#### Hydrogen Peroxide Content

Estimation of H_2_O_2_ content was done by the protocol of Velikova et al. (2000). Fresh seedlings were macerated in 2 ml of 0.1% trichloroacetic acid (TCA) followed by centrifugation at 12,000 rpm for 15 min. Of the supernatant, 0.4 ml was mixed with an equal volume of 10 mM potassium phosphate buffer, pH 7.0. Potassium iodide (0.8 ml) was added to the mixture. The absorbance of the reaction mixture was read at 390 nm. The concentration of H_2_O_2_ was determined the standard curve of H_2_O_2_ and expressed as μmol g^–1^ FW.

#### Malondialdehyde Content

MDA content was quantified following the protocol of Heath and Packer (1968). One gram of fresh seedlings was extracted in 3 ml of 0.1% TCA and then centrifuged at 12,000 rpm for 15 min in chilled conditions. Following centrifugation, 4 ml 0.5% thiobarbituric in 20% TCA acid was added to the collected supernatant. The mixture was heated at 95°C for 30 min and immediately cooled for termination of reaction by keeping it on ice bath. The absorbance of the colored complex was taken at 532 and 600 nm. MDA content was calculated by taking the difference in absorbance using an extinction coefficient of 155 mM^–1^ cm^–1^ and expressed as μM g^–1^ FW.

### *In situ* Visualization of Superoxide Anion, Hydrogen Peroxide, Lipid Peroxidation, Cell Viability, and Membrane and Nuclear Damage

#### Histochemical Detection of Superoxide Anion and Hydrogen Peroxide

Histochemical detection of O_2_•^–^ and H_2_O_2_ was accomplished using nitroblue tetrazolium (NBT) and 3,3′-diaminobenzidine (DAB) following the methods of Thordal-Christensen et al. (1997) and Frahry and Schopfer (2001), respectively. Cotyledons of the seedlings were immersed in NBT and DAB followed by boiling in ethanol to clearly visualize blue and brown spots, respectively. In *B. juncea* roots, H_2_O_2_ was tagged with 2′,7′-dichlorofluorescin diacetate (DCF-DA) following the method given by Rodríguez-Serrano et al. (2009). Root tips were stained by immersing in 25 μM DCF-DA for 30 min in dark. Following staining, the roots were washed with distilled water and mounted on glass slides and observed under a fluorescent microscope at an excitation wavelength of 488 nm and an emission wavelength of 530 nm.

#### Lipid Peroxidation and Cell Viability

*In situ* visualization of lipid peroxidation and cell viability was done using Schiff’s reagent and Evans’s blue following the method given by Pompella et al. (1987) and Yamamoto et al. (2001), respectively. Cotyledons of the seedlings were engrossed in Schiff’s reagent and Evans’s blue for 20 min. Afterward, the cotyledons were bleached by immersing them in boiling ethanol. Cotyledons were placed on glass slides and photographed.

#### Fluorescent Imaging of Membrane and Nuclear Damage

The membrane and nuclear damages were visualized using fluorescent dyes propidium iodide (PI) and 4,6-diamino-2-phenylindole (DAPI) according to the method of Callard et al. (1996) and Gutiérrez-Alcalá et al. (2000), respectively. For *in situ* visualization of membrane damage, the roots of *B. juncea* seedlings were soaked in PI (50 μM, Sigma-Aldrich, Mumbai, India) for 15 min followed by washing with distilled water. Roots were mounted on glass slides and visualized under a Ti2 fluorescent microscope at an excitation wavelength of 543 nm and emission wavelength of 617 nm. For visualization of nuclear damage, the roots were stained using DAPI and incubated in dark for 30 min followed by rinsing with PBS and finally mounted on slides and observed under fluorescent microscope at an excitation wavelength of 358 nm and emission wave length of 461 nm.

#### Determination of the Activities of Antioxidant Enzymes

To assess the activities of various antioxidant enzymes and determine protein content, 1 g of fresh plant sample was homogenized in 3 ml of 50 mM potassium phosphate buffer (PPB) having pH 7.0 in a pre-chilled pestle mortar. To assess the SOD activity, 50 mM sodium carbonate buffer having pH 10.2 was used for crushing the plant sample. The homogenate was centrifuged at 12,000 rpm at 4°C for 20 min. The supernatant was used to assess the activities of antioxidant enzymes and protein content. Determination of protein content was done following the method given by Bradford (1976). Protein content was calculated using the standard curve of bovine serum albumin.

#### Superoxide Dismutase (SOD EC. 1.15.1.1)

The activity of SOD was assessed by the protocol given by Kono (1978). The reaction mixture comprised of 50 mM sodium carbonate (Na_2_CO_3_) buffer, 0.03% Triton X-100, 1 mM hydroxylamine hydrochloride (pH 6), 24 μM nitroblue tetrazolium (NBT), and 0.1 M ethylenediaminetetraacetic acid (EDTA) and plant extract. Reaction mixture was incubated for 2 min, and the absorbance was taken at 540 nm for 1 min. Activity of SOD was determined by computing the inhibition of NBT. SOD unit activity is defined as the quantity of enzyme required for 50% inhibition. Specific activity was expressed as units per minute per milligram of the protein.

#### Guaiacol Peroxidase (POD EC 1.11.1.7)

Activity of POD was assessed by the method of Pütter (1974). The reaction mixture included the plant extract, 50 mM of potassium phosphate buffer (pH 7.0), 20.1 mM guaiacol solution, and 123 mM H_2_O_2_. The increase in absorbance due to the formation of guaiacol dehydrogenation product was read at 436 nm for 1 min using ε = 26.6 mM^–1^ cm^–1^.

#### Catalase (CAT EC 1.11.1.6)

The activity of CAT was determined by the procedure described by Aebi (1984). The reaction mixture contained plant extract, 50 mM phosphate buffer pH 7.0), and15 mM H_2_O_2_. The decrease in absorbance due to decomposition of H_2_O_2_ was measured at 240 nm for 1 min using ε = 39.4 mM^–1^ cm^–1^.

#### Ascorbate Peroxidase (APOX EC 1.11.1.11)

The activity of APOX was assessed by following the protocol mentioned by Nakano and Asada (1981). The reaction mixture comprised of 50 mM phosphate buffer (pH 7.0), 0.5 mM ascorbate, 1 mM H_2_O_2_, and enzyme extract. Decline in absorbance was read at 290 nm for 1 min by using extinction coefficient of 2.8 mM^–1^ cm^–1^.

#### Dehydroascorbate Reductase (DHAR EC 1.8.5.1)

Activity of DHAR was assessed by protocol presented by Dalton et al. (1986). The reaction mixture comprised of 50 mM phosphate buffer (pH 7.0), enzyme extract, 0.1 mM EDTA, 0.2 mM dehydroascorbate, and 1.5 mM reduced glutathione (GSH). The change in absorbance was read at 265 nm for 1 min by using an extinction coefficient of 14 mM^–1^ cm^–1^.

#### Glutathione Reductase (GR EC 1.6.4.2)

Activity of GR was determined by the method described by Carlberg and Mannervik (1975). The reaction mixture comprised of 50 mM phosphate buffer (pH 7.0), enzyme extract, 0.1 mM nicotinamide adenine dinucleotide phosphate reductase (NADPH), 1 mM glutathione disulfide (GSSG), 1 mM EDTA, and the plant extract. The decrease in absorbance was measured at 340 nm for 1 min by using an extinction coefficient of 6.22 mM^–1^ cm^–1^.

#### Glutathione Peroxidase (GPOX EC 1.11.1.9)

Activity of GPOX was determined by the method given by Flohé and Günzler (1984). The reaction mixture comprised of the plant extract, 50 mM phosphate buffer pH (7.0), 1 mM sodium azide, 0.5 mM EDTA, 1 mM reduced glutathione, 0.15 mM NADPH, and 0.15 mM H_2_O_2_. The decline in absorbance was measured at 340 nm for 1 min by using an extinction coefficient of 6.22 mM^–1^ cm^–1^.

#### Glutathione-S-Transferase (GST EC 2.5.1.18)

The activity of GST was assessed by the procedure described by Habig et al. (1974). The reaction mixture comprised of 0.1 M phosphate buffer (pH 7.4), 1 mM 2,4 dinitrochlorobenzene (cDNB), 20 mM GSH, and the plant extract. The absorbance was read at 340 nm by using an extinction coefficient of 9.6 mM^–1^ cm^–1^.

### Estimation of Non-enzymatic Antioxidants

For the estimation of non-enzymatic antioxidants, 1 g of fresh plant sample was homogenized in 50 mM Tris buffer (pH 10) and centrifuged at 13,000 rpm for 20 min at 4°C. The supernatant was used for measurement of non-enzymatic antioxidants.

#### Ascorbic Acid

Ascorbic acid content was estimated by following the protocol of Roe and Kuether (1943). Plant extract (0.5 ml) was mixed with 0.5 ml of 50% trichloroacetic acid (TCA). Activated charcoal (100 mg) and 4 ml of double-distilled water was added to the mixture. The mixture was thoroughly mixed and filtered using Whatman filter paper No. 1. The filtrate was collected, and 0.4 ml of 2,4- dinitrophenyl hydrazine (DNPH) was added. The reaction mixture was incubated at 37°C for 3 h. Chilled sulfuric acid (65%) (1.6 ml) was added, and the mixture was cooled at room temperature for 30 min, and the absorbance was measured at 520 nm. Ascorbic acid (1 mg/100 ml) was used as the standard.

#### Glutathione

The glutathione content was estimated following the method proposed by Sedalk and Lindsay (1968). The reaction mixture containing 100 μl of plant extract, 50 μl of Ellman’s reagent, 1 ml of Tris buffer, and 4 ml of absolute ethanol was incubated at room temperature for 15 min. The reaction mixture was centrifuged at 3,000 rpm for 15 min. The absorbance of the supernatant was recorded at 412 nm. GSH (1 mg/100 ml) was used as standard.

#### Fluorescent Tagging of Glutathione

Glutathione tagging was done in roots following the method of Fricker and Mayer (2001). The roots were stained with 25 μM monochlorobimane (MCB) containing 5 μM sodium azide for 15–20 min. After incubation, roots were washed with distilled water, and images were obtained with a fluorescent microscope at excitation wavelength of 351–364 nm and emission wavelength of 477 nm.

#### Determination of Total Antioxidant Capacity

Total lipid- and water-soluble antioxidant capacity was determined by an antioxidant analyzer (PHOTOCHEM BU, Analytik Jena, Germany). One gram of fresh plant sample was extracted in 5 ml 50 mM Tris (pH 10.0) for water-soluble antioxidants and 1.5 ml methanol for lipid-soluble antioxidants under chilled environment, followed by centrifugation at 13,000 rpm at 4°C for 20 min. The antioxidant capacity was determined using standard kits and instructions provided in the manual available with the antioxidant analyzer. Total lipid- and water-soluble antioxidants were expressed in micromoles per gram.

### Analysis of Gene Expression

#### RNA Isolation and Preparation of cDNA

RNA isolation from *B. juncea* seedlings was done using Trizol (Invitrogen, Waltham, MA, United States). It was quantified using a Nanodrop spectrophotometer (Thermo Fisher Scientific, Waltham, MA, United States). Qualitative analysis of RNA was done on 2% agarose gel electrophoresis. The isolated RNA was incubated with DNase (Ambion TURBO DNA free, Life Technologies, Carlsbad, CA, United States) to eliminate any potential DNA impurity. The first-strand of the cDNA was prepared from 1 μg of DNAse-treated RNA using reverse transcriptase (Promega, Madison, WI, United States) with oligo (dt) primer.

#### Expression Analysis

The qRT-PCR-based quantification was done in triplicates and conducted on ROTOR-GENE Q RT-PCR system (Qiagen, United States) following the manufacturer’s instructions. Reaction mixture consisted of SYBER Green Master mix, diluted cDNA, and specific primer for the genes designed with Primer 3 software (Table 1) and cDNA. The conditions for qRT-PCR were 95°C for 10–12 min for initial denaturation, followed by 40 cycles of three steps of amplification 95°C (10 s), 66°C (10 s), and 72°C (15 s). All reactions were performed in triplicates using gene-specific primers and selecting actin gene as reference gene to normalize the data, and threshold cycle (Ct) values were used for calculation. Quantification of the relative gene expression of a gene was done by using 2^–ΔΔct^ method (Livak and Schmittgen, 2001; Awasthi et al., 2015).

**TABLE 1:** Sequences of the gene specific primers for used for qRT-PCR.

Gene	Primer sequence
*Actin*	Forward Primer 5′ ACTGGTATTGTGCTTGACTCTG3′ Reverse Primer 5′ AGCTTCTCTTTAATGTCACGGAC3′
*SOD*	Forward Primer 5′ CACATTTCAACCCTGATGGTAA3′ Reverse Primer 5′ACAGCCCTTCCGACAATA3′
*POD*	Forward Primer 5′TTCGAACGGAAAAAGATGCT3′ Reverse Primer 5′AACCCTCCATGAAGGACCTC3′
*CAT*	Forward Primer 5′GTTCGACTTTGACCCACT3′ Reverse Primer 5′ATCCCAGGAACAATGATAGC3′
*APOX*	Forward Primer 5′CCACTTGAGACAGGTGTTACTA3′ Reverse Primer 5′TCCTTGAAGTAAGAGTTGTCGAAA3′
*DHAR*	Forward Primer 5′CTGGATGAGCTTAGTACATTCAAC3′ Reverse Primer 5′GGAAAGAAAGTGAATCTGGAACA3′
*GR*	Forward Primer 5′GATGCAGCGCTTGATTTAC3′ Reverse Primer 5′TCCCTAACGTCTTCATCAAACC3′
*GST*	Forward Primer 5′GAGCACAAGAAAGAGCCC3′ Reverse Primer 5′TGTTCTTGGAGTCGGCTG 3′
*RBOH*	Forward Primer 5′ACGGGGTGTGATAGAGATGC3′ Reverse Primer 5′TTTTTCCAGTTGGGTCTTGC3′

### Statistical Analysis

The experiments were performed in triplicates, and the results presented are in means ± SE in figures. Statistical analysis was performed using one-way analysis of variance (ANOVA) using SPSS 16.0 followed by Tukey’s honestly significant difference (HSD) test to determine significant differences among means of the treatments at 5% level of significance.

## Results

### Effect of Earthworms and Plant-Growth-Promoting Rhizobacteria on Biomass of *Brassica juncea* Seedlings Under Chromium Stress

**FIGURE 1:**
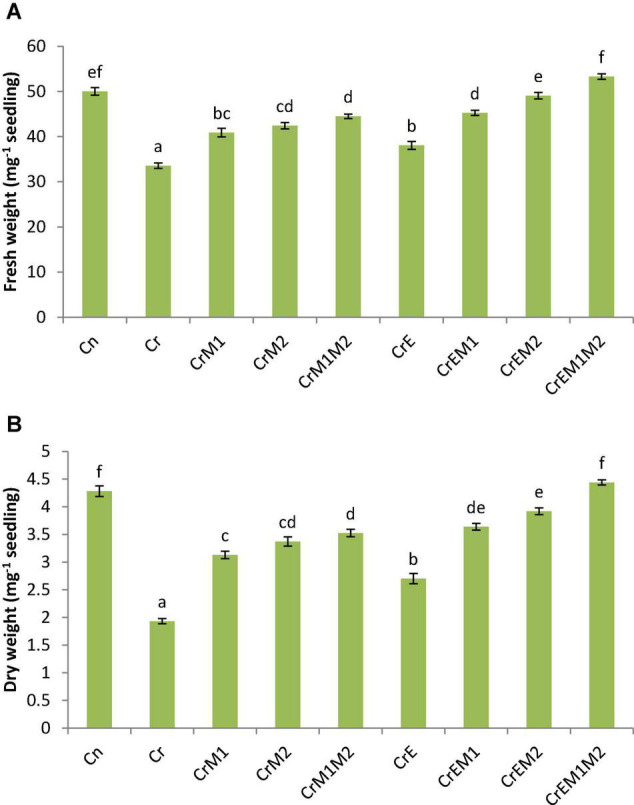
Effect of PGPR [*Pseudomonas aeruginosa* (M1) & *Burkholderia gladioli* (M2)] and the earthworms [*Eisenia fetida* (E)] on **(A)** fresh weight and **(B)** dryweight of 10-day-old *B. juncea* seedlings under chromium (Cr) toxicity. Data are presented as means of three independent replicates ± S.E. (standard error). Different letterson the bar graphs indicate that mean values of the treatments are significantly different at *p* ≤ 0.05 (Tukey’s test).

To assess the effect of earthworms and PGPR inoculation on biomass of *B. juncea* under Cr stress, fresh and dry weights were determined in terms of fresh weight and dry weight (Figures 1A,B). Cr exposure to the seedlings significantly reduced the fresh weight of the seedlings by 32.91% and dry weight by 54.86% in comparison to untreated control. However, supplementation of earthworms and PGPR (M1 and M2) alone and in combinations significantly improved the seedlings biomass. Supplementation of earthworms enhanced the fresh and dry weights by 13.42 and 39.85% when compared with Cr only. Simultaneously, inoculation of M1, M2, and M1M2 under Cr stress also significantly enhanced the biomass of the seedlings when compared with Cr treatment only. Dual inoculation of both PGPR along with earthworms under Cr contamination resulted in highest significant increase in fresh weight by 58.82% and dry weight by 129.69 as compared with sole Cr-treated seedlings. The results depict that Cr had a negative impact on the seedling biomass, which was improved by supplementation with earthworms and PGPR showing that earthworms and PGPR promote the biomass.

### Effect of Earthworms and Plant-Growth-Promoting Rhizobacteria on Oxidative Stress in *Brassica juncea* Seedlings Under Chromium Stress

**FIGURE 2:**
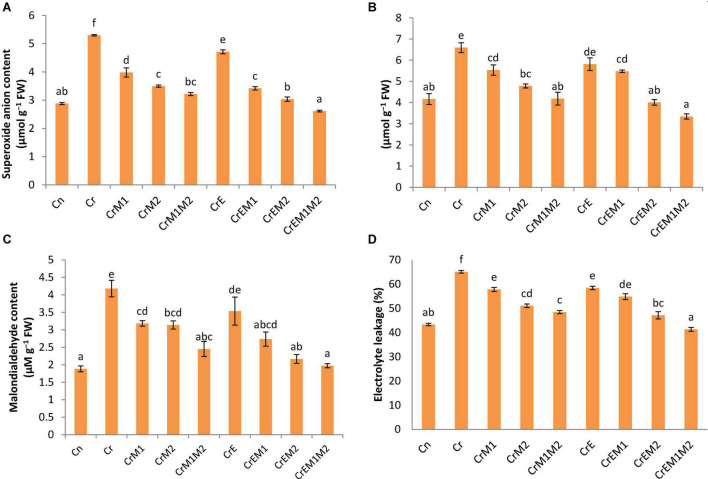
Effect of PGPR [*Pseudomonas aeruginosa* (M1) & *Burkholderia gladioli* (M2)] and earthworms [*Eisenia fetida* (E)] on **(A)** superoxide anion, **(B)** hydrogenperoxide, **(C)** MDA content, and **(D)** electrolyte leakage in 10-day-old *B. juncea* seedlings, under chromium (Cr) stress. Data are presented as means of three independent replicates ± S.E (standard error). Different letters on the bar graphs indicate that mean values of the treatments are significantly different at *p* ≤ 0.05 (Tukey’s test).

*Brassica juncea* seedlings exposed to Cr unveiled high levels of oxidative stress due to enhanced production of ROS, *viz.*, O_2_•^–^ and H_2_O_2_. A severe rise in the level of O_2_•^–^ by 84.32% and H_2_O_2_ by 58.41% in Cr-treated seedlings was revealed in comparison to control seedlings. Diminution in oxidative stress in Cr-treated seedlings was revealed with sole earthworms and PGPR inoculation and their combined applications as compared to only Cr-treated seedlings. However, a steep decline in O_2_•^–^ content by 50.59% (Figure 2A) and H_2_O_2_ content by 49.27% (Figure 2B) was observed in seedlings raised with the combined inoculation of earthworms along with both PGPR (M1M2).

**FIGURE 3:**
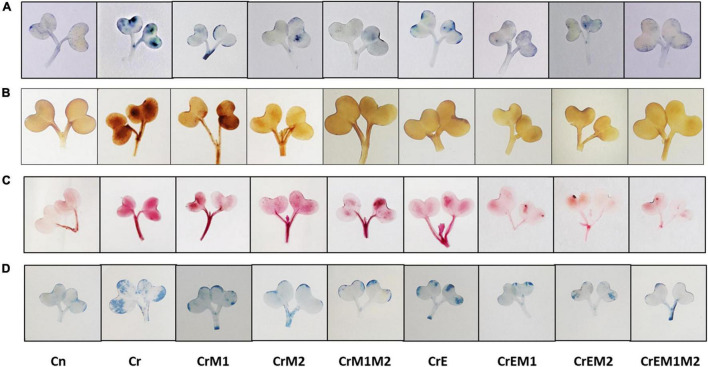
*In vivo* imaging of **(A)** superoxide anion (O_2_•^–^) stained with NBT, **(B)** hydrogen peroxide (H_2_O_2_) detected using DAB, **(C)** lipid peroxidation, asMDA content stained using Schiff’s reagent, and **(D)** cell viability visualized using Evan’s blue in *B. juncea* seedlings, under Cr stress.

**FIGURE 4:**
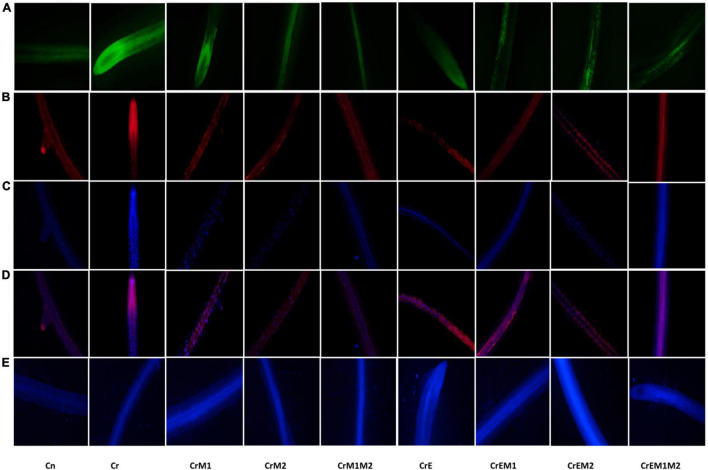
*In vivo* fluorescence visualization of **(A)** hydrogen peroxide (H_2_O_2_) stained with DCF-DA, **(B)** membrane damage detected using PI, **(C)** nuclear damagemarked with DAPI, **(D)** composite of PI and DAPI, and **(E)** glutathione tagged with MCB.

The spectroscopic observations obtained for O_2_•^–^ and H_2_O_2_ content are in conformity with their histochemical detection in cotyledons with NBT and DAB, respectively. Blue spots and dark brown patches portray the accumulation of O_2_•^–^ and H_2_O_2_ (Figures 3A,B), respectively, in *B. juncea* cotyledons under Cr treatment. However, supplementation of earthworms and PGPR alone and in combinations reduced the spots on the cotyledons produced by O_2_•^–^ and H_2_O_2_ signifying reduction in ROS accumulation compared to Cr stress. In addition, the annotations obtained from fluorescent tagging of H_2_O_2_ with 2,7-dichloroflourcein diacetate (DCF-DA) in roots of *B. juncea* seedlings showed bright fluorescent green color under Cr stress (Figure 4A). However, roots of seedlings grown in control and in combinations with earthworms and PGPR exhibited the faint green appearance of DCF suggesting the ameliorative role of earthworms and PGPR in busting ROS.

### Effect of Earthworms and Plant-Growth-Promoting Rhizobacteria on Lipid Peroxidation, Electrolyte Leakage, and Cell Viability of *Brassica juncea* Seedlings Under Chromium Stress

Lipid peroxidation in seedlings was measured as MDA content. *Brassica juncea* seedlings raised in Cr-contaminated soils showed a sharp increase in the MDA content (122.34%) and electrolyte leakage (50.19%) as compared to treatment without Cr. However, inoculation of earthworms to Cr-treated seedlings reduced the MDA content and electrolyte leakage by 15.41 and 10.22%, respectively, as compared with the Cr-stressed seedlings. MDA and electrolyte leakage showed maximum reduction by 52.77 and 36.48%, respectively, when inoculated with earthworms and M1M2 (Figures 2C,D). The results of *in situ* histochemical visualization of lipid peroxidation reagent and cell viability using Schiff’s reagent and Evan’s blue, respectively, in cotyledons of *B. juncea* are depicted in Figures 3C,D. Seedlings grown in Cr only showed a dark pink hue indicator of lipid peroxidation (Figure 3C), and a deep blue color signifies loss of cell viability (Figure 3D), which, on inoculation with earthworms and PGPR, showed a light staining pattern, indicating improved lipid peroxidation and cell viability. Membrane damage and damages were visualized *in situ* through a fluorescent microscope using PI and DAPI showing accumulation of red and blue color, as a measure of membrane and nuclear damage, respectively, in a concentration-dependent manner. The inoculation of earthworms alone or in the presence of PGPR led to appreciable lowering of red and blue color, which showed reduced membrane and nuclear damages. Earthworms and PGPR play an imperative role in mitigating Cr toxicity by lowering the MDA content and electrolyte leakage, which improved lipid peroxidation, prevented membrane and nuclear damages, and reinstated cell viability.

### Effect of Earthworms and Plant-Growth-Promoting Rhizobacteria on Antioxidant Enzyme Activity of *Brassica juncea* Seedlings Under Chromium Stress

To assess the effect of earthworms and PGPR on various antioxidant enzymes, activities of various antioxidant enzymes were evaluated. Table 2 shows that Cr treatment modulated the activities of these enzymes. The activities of SOD, POD, APOX, GPOX, DHAR, GR, and GST were enhanced by 70.16, 32.99, 41.49, 116.76, 88.99, 60.01, and 51.51% in *B. juncea* in response to Cr treatment, while the activity of CAT was found to be declined by 26.69% as compared to control. Inoculation of earthworms along with PGPR further enhanced the activities of SOD, POD, CAT, APOX, DHAR, GR, and GST in seedlings under Cr stress. However, a decline in GPOX activity was observed upon inoculation with PGPR and earthworms. The maximum increase in activities of SOD, POD, CAT, APOX, DHAR, GR, and GST was noticed in seedlings grown in Cr stress supplemented with earthworms along with combined inoculation of M1M2 by 118.79, 63.48, 104.43, 222.23, 217.12, 69.80, and 82.45%, respectively, in comparison to the seedlings treated with Cr only. The activity of GPOX declined extremely by 82.46% with the supplementation of earthworms along with dual inoculation of M1M2.

**TABLE 2:** Effect of PGPR [*Pseudomonas aeruginosa* (M1) & *Burkholderia gladioli* (M2)] and the earthworms [*Eisenia fetida* (E)] on the activity of SOD, POD, CAT, APOX, DHAR, GR, GPOX, GST in 10 day old *B. juncea* seedlings under chromium (Cr) toxicity.

Treatments	SOD	POD	CAT	APOX	DHAR	GR	GPOX	GST
Cn	11.17 ± 1.55^a^	23.79 ± 1.02 ^a^	24.94 ± 0.97^a^	8.41 ± 0.95^a^	6.14 ± 0.77^a^	7.03 ± 0.11 ^a^	12.04 ± 0.15 ^a^	10.13 ± 0.43^a^
Cr	19.01 ± 1.92^b^	31.65 ± 1.11^b^	18.28 ± 0.47^ab^	11.90 ± 1.54^a^	11.61 ± 0.84^ab^	11.24 ± 0.39 ^b^	26.10 ± 0.67^e^	15.36 ± 1.04^b^
CrM1	27.74 ± 1.77^cd^	43.94 ± 1.06 ^d^	25.88 ± 1.39 ^cd^	19.99 ± 1.00^b^	21.12 ± 1.11^cd^	13.93 ± 0.54^cd^	17.82 ± 0.52 ^cd^	20.25 ± 0.98^cd^
CrM2	29.69 ± 0.88^cd^	45.27 ± 1.40^de^	29.84 ± 1.36 ^cd^	22.80 ± 1.11^bc^	23.49 ± 1.08d^e^	16.31 ± 0.29^e^	17.73 ± 0.57 ^cd^	21.17 ± 0.62^cd^
CrM1M2	33.62 ± 1.25^e^	49.54 ± 1.98^def^	32.46 ± 1.52^ef^	26.04 ± 0.56^c^	27.34 ± 1.42^de^	15.95 ± 0.77 ^de^	14.59 ± 0.62^ab^	24.17 ± 0.39^def^
CrE	26.15 ± 1.94^bc^	39.28 ± 0.66^c^	23.86 ± 0.98^bc^	20.06 ± 0.92^b^	15.34 ± 1.36^bc^	12.73 ± 0.69^bc^	20.26 ± 0.75^d^	18.38 ± 0.52^bc^
CrM1E	3 4.05 ± 1.26 ^de^	46.20 ± 1.25^def^	29.24 ± 0.93^cde^	33.07 ± 1.24^d^	24.87 ± 0.89^ef^	16.24 ± 0.45^e^	15.67 ± 0.3^bc^	23.43 ± 0.65^de^
CrM2E	38.93 ± 1.02^ef^	49.89 ± 0.70^ef^	34.12 ± 0.78^ef^	35.46 ± 1.11^de^	30.97 ± 1.69^fg^	19.09 ± 0.25^f^	12.13 ± 0.4^a^	26.08 ± 1.30^ef^
CrM1M2E	41.61 ± 1.29^f^	51.74 ± 0.46 ^f^	37.37 ± 0.65^f^	38.35 ± 0.82^e^	36.82 ± 1.30^g^	16.53 ± 0.18^e^	12.09 ± 0.20^a^	28.02 ± 1.56^f^

*Data is presented as means of 3 replicates ± S.E (standard error). Different letters in the table indicate that mean values of the treatments are significantly different at (p ≤ 0.05, Tukey’s test).*

### Effect of Earthworms and Plant-Growth-Promoting Rhizobacteria on Total Antioxidant Capacity and Non-enzymatic Antioxidants of *Brassica juncea* Seedlings Under Chromium Stress

**FIGURE 5:**
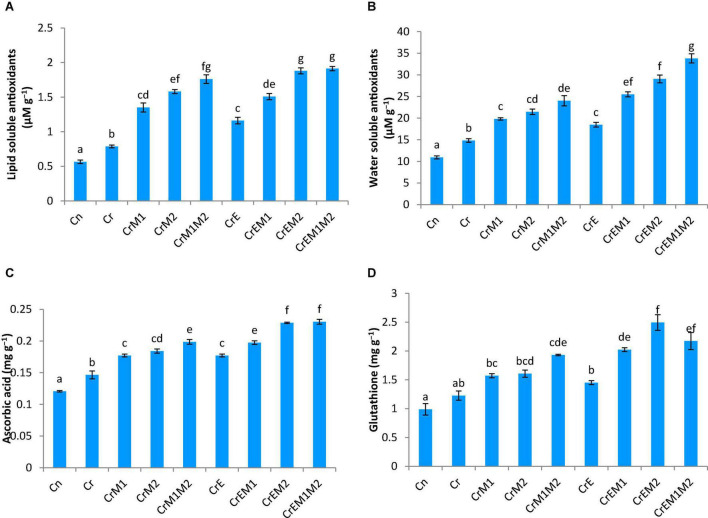
Effect of PGPR [*Pseudomonas aeruginosa* (M1) & *Burkholderia gladioli* (M2)] and earthworms [*Eisenia fetida* (E)] on **(A)** lipid soluble antioxidants, **(B)** watersoluble antioxidants, **(C)** ascorbic acid, and **(D)** glutathione content in 10-day-old *B. juncea* seedlings, under chromium (Cr) toxicity. Data are presented as means of 3 independent replicates ± S.E (standard error). Different letters on the bar graphs indicate that mean values of the treatments are significantly different at *p* ≤ 0.05 (Tukey’s test).

Total antioxidant content was enhanced under Cr stress. Concentration of water-soluble antioxidants was enhanced by 35.69% and that of lipid-soluble antioxidants got increased by 47.56% under Cr stress in comparison to control. The addition of earthworms to Cr-treated seedlings further enhanced the levels of water- and lipid-soluble antioxidants by 24.60 and 47.56%, respectively. M1 and M2 treatment along with earthworms further enhanced water- and lipid-soluble antioxidants by 128.11 and 143.03%, respectively, as compared to the seedlings grown in Cr only (Figures 5A,B). Non-enzymatic antioxidants like ascorbic acid and glutathione content were observed to be increased in Cr-stressed seedlings, and inoculation of earthworms and PGPR further boosted their activity. A tremendous increase of 21.4% in ascorbic acid content was observed in Cr stressed seedlings as compared to control, which was further promoted by 56.07% with earthworms and M2 treatment (Figure 5C). Glutathione content was increased by 24.09% under Cr stress relative to control seedlings. Amendment of Cr-stressed seedlings with earthworms and M2 enhanced the glutathione content by 103.28% (Figure 5D). Intracellular glutathione in root sections were tagged using non-fluorescent probe MCB. MCB binds with glutathione and other low molecular weight thiols to form a fluorescent blue conjugate, glutathione-S-bimane (GSB). Fluorescent microscopy suggested that roots of Cr-stressed seedlings showed an intensive blue color, which shows enhanced levels of glutathione as compared to the control (Figure 4D). The intensity of blue color was further enhanced in Cr-stressed seedlings inoculated with earthworms along with PGPR.

### Effect of Earthworms and Plant-Growth-Promoting Rhizobacteria on Expression of Key Genes of Antioxidant Enzymes of *Brassica juncea* Seedlings Under Chromium Stress

**FIGURE 6:**
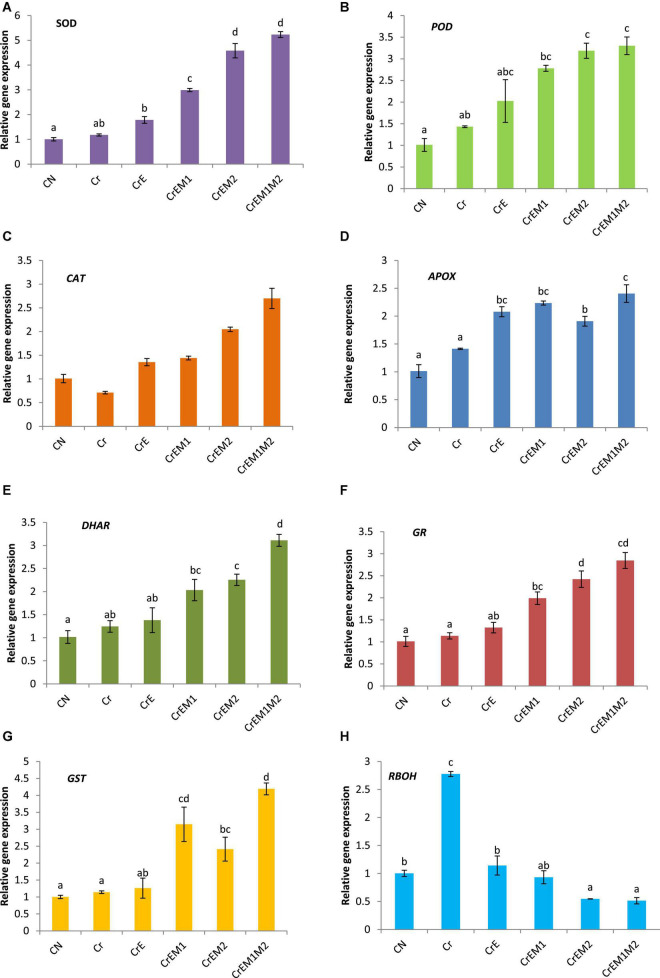
Effect of PGPR [*Pseudomonas aeruginosa* (M1) & *Burkholderia gladioli* (M2)] and earthworms [*Eisenia fetida* (E)] on the gene expression of **(A)** SOD, **(B)** POD, **(C)** CAT, **(D)** APOX, **(E)** DHAR, **(F)** GR, **(G)** GST, and **(H)** RBOH in 10-day-old *B. juncea* seedlings, under chromium (Cr) stress. Data are presented as means of 3 independent replicates ± S.E (standard error). Different letters on the bar graphs indicate that mean values of the treatments are significantly different at *p* ≤ 0.05 (Tukey’s test).

The expression analysis of key genes of antioxidant enzymes, *viz.*, SOD, POD, CAT, APOX, DHAR, GR, and GST, at the transcript level was carried out in Cr-stressed *B. juncea* seedlings, which revealed the upregulation of these genes under Cr stress as compared to control. The reduction in CAT activity was noticed under Cr stress as compared to control. Supplementation of earthworms alone and along with PGPR (M1, M2 alone, and their binary combination, i.e., M1M2) enhanced the expression of the aforementioned enzymes in *B. juncea* seedlings grown under Cr stress as compared to Cr treatment only. The maximum augmentation in the expression of *SOD* (4.44− fold), *POD* (2.30-fold), *CAT* (3.79-fold), *APOX* (1.70-fold), *DHAR* (2.48-fold), *GR* (2.50-fold), and *GST* (3.67-fold) (Figures 6A–G) was observed in Cr-stressed seedlings treated with earthworms along with binary combination of M1 and M2, i.e., EM1M2 when compared to Cr-stressed seedlings only. Furthermore, upregulation of the RBOH1 (respiratory burst oxidase 1) gene in Cr-stressed *B. juncea* seedlings was noticed in comparison to control. Maximum reduction of 0.185-fold in the expression of RBOH1 was noticed with the addition of earthworms along with M1M2, i.e., EM1M2 in Cr-stressed *B. juncea* seedlings in comparison to Cr stress only (Figure 6H).

### Correlation Analysis

**FIGURE 7:**
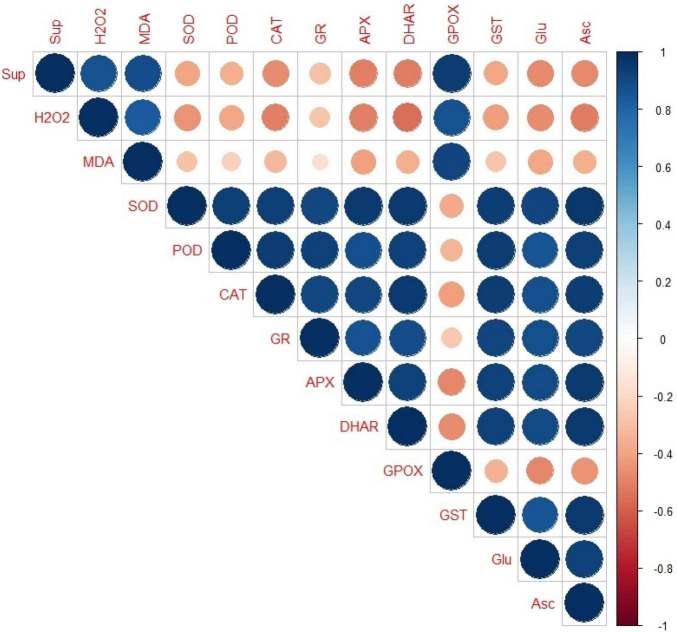
Corr-plot representing the Pearson’s correlation analysis between the oxidative stress markers and oxidative defence molecules (enzymatic andnon-enzymatic). Here, the size of the circles is proportional to the absolute value of correlation coefficients, whereas their colour represents the value in positive or negative correlation region as represented in the right bar of varying colour intensities from intense blue to intense red, ranging from +1 to −1, respectively. Sup, superoxide anion; H_2_O_2_,hydrogen peroxide; MDA, malondialdehyde; SOD, superoxide dismutase; POD, guaiacol peroxidase; CAT, catalase; GR, glutathione reductase; APX/APOX, ascorbate peroxidase; DHAR, dehydroascorbate reductase; GPOX, glutathione peroxidase; GST, glutathione-S-transferase; Glu, glutathione; and Asc, ascorbic acid.

The Corr plot was made (Figure 7) using based on Pearson’s correlation analysis between the oxidative stress markers and oxidative defense molecules (both enzymatic and non-enzymatic). It was found that except GPOX, all the antioxidant enzymes (SOD, POD, CAT, GR, APX/APOX, DHAR, GPOX, and GST) and the non-enzymatic antioxidants (Glu and Asc) had a strong positive correlation among themselves but a week negative correlation with the two ROS species, i.e., superoxide anion and H_2_O_2_ and the MDA content.

## Discussion

Plants imperiled to heavy metal stress have harmful effects on various metabolic processes due to generation of ROS resulting in reduced plant biomass, production, and yield. Disproportionate generation of ROS has deleterious effects on various cellular components, which affect the cellular integrity, leading to cell death (Askari et al., 2021). Cr stress has damaging effect on plant biomass and augments the production of ROS that hinders the function of the plant (Qureshi et al., 2020). Soil micro- and macroorganisms can be reconnoitered as plant growth promoters to diminish the adverse effect of Cr stress *via* the modulation of enzymatic and non-enzymatic antioxidants. The present study demonstrated the efficacy of PGPR and earthworms in ameliorating Cr-induced oxidative damage in *B. juncea* seedlings *via* production of enzymatic and non-enzymatic antioxidant molecules. In the present investigation, Cr stress significantly affected the bio-mass of *B. juncea* seedlings as indicated by reduction in fresh and dry weights of seedlings (Figures 1A,B). Cr toxicity has also been shown to reduce biomass in *B. juncea* (Mahmud et al., 2017), *S. seban* (Din et al., 2020), tomato, and brinjal (Singh and Prasad, 2019). The reduction in biomass in Cr-stressed seedlings could be attributed to the excessive production of ROS (Figures 2A,B) and reduced water and nutrient uptake. According to several studies, PGPR enhances the nutrient uptake and secrete various plant-growth-promoting metabolites in the rhizosphere, which aid in iron and phosphate solubilization. Furthermore, they may also release phytohormones, which restore plant growth and biomass in stressed conditions (Cai et al., 2019; Shreya et al., 2020). Earthworms promote plant growth by improving soil structure and enhancing the nutrient availability and microbial activity in rhizosphere (Kaur et al., 2017). The growth-promoting role of earthworms has been reported by Jusselme et al. (2012). Concomitantly, Gupta et al. (2020) documented that *Enterobacter* sp. and *Klebsiella* sp. improved the biomass of tomato plant under Cr stress. Our results indicated that inoculation of PGPR and earthworms to the stressed seedlings considerably improved the fresh and dry weights of the seedlings.

Implication of Cr causes the excessive generation of ROS due to altered redox status and antioxidant defense system leading to the cellular condition considered as oxidative stress. Overgeneration of ROS damages the cellular macromolecules, which results in lipid peroxidation destroying membrane integrity and causing cell death (Sharma et al., 2019; Singh and Prasad, 2019). In the present study, the Cr-induced oxidative stress was revealed as the overproduction of O_2_•^–^ and H_2_O_2_ in *B. juncea* seedlings, which is evident from their concentration at cellular levels (Figures 2A,B) and results from histochemical studies (Figures 3A,B, 4A), which is also in agreement with the biochemical data. The present study also revealed the upregulation of respiratory burst oxidase homolog gene in Cr-stressed plants as compared to control (Figure 6H). In plants, ROS bursts take place through NADPH oxidase/respiratory burst oxidase homolog (RBOH 1) proteins [responsible for superoxide generation) under various abiotic stress conditions (Kaur et al., 2014; Chapman et al., 2019)]. A sudden increase in ROS accumulation was also observed previously by Handa et al. (2018) in *B. juncea* seedlings grown under Cr stress. The results of the present study revealed the synergistic interactions of earthworms with both PGPR in successfully ameliorating oxidative stress in *B. juncea* seedlings under Cr stress. However, supplementation of both M1 and M2 along with earthworms significantly abridged ROS accumulation by downregulating the expression of gene for ROS production (*RBOH1* for O_2_•^–^ production). Our results are in alliance with Wang et al. (2020) who showed that *Sphingomonas* SaMR12 inoculation to *B. juncea* under Cd stress lowered O_2_•^–^ and H_2_O_2_ content. Cr application to *B. juncea* seedlings also escalated the accumulation MDA (Figure 2C), which is produced as a result of peroxidation of membrane lipids and prominent indicator of membrane damage due to oxidative stress. MDA damages cell by reacting with free amino groups and disrupting inter- and intramolecular cross-linkages of proteins (Islam et al., 2016). The elevated levels of MDA have also been reported in *Arabidopsis thaliana*, *Zea mays*, and *Brassica napus* under Cr stress (Afshan et al., 2015; Anjum et al., 2017; Ding et al., 2019). Histochemical data of ROS and their concomitant damage to lipids and membranes depicted that Cr toxicity elevated their content in seedlings (Figure 3C). The magnitude of membrane and nuclear damages caused due to Cr toxicity in *B. juncea* roots was also corroborated using fluorescence microscopy (Figures 4B,C). Decreased lipid peroxidation was documented upon the inoculation of *Klebsiella* sp. and *Enterobacter* sp. to *Helianthus annuus* exposed to Cr stress (Gupta et al., 2020). The reduced level of MDA observed upon inoculation with PGPR might owe to altered antioxidant defense system of the plants (Janmohammadi et al., 2013). Concurrently, supplementation of *E. fetida* to Cd-stressed *B. juncea* seedlings lowered the O_2_•^–^, H_2_O_2_, and MDA level (Kaur et al., 2019). Lipid peroxidation also aggravates electrolyte leakage in Cr-stressed *B. juncea* seedlings (Figure 2D) leading to reduced cell viability (Figure 3D) and enhanced membrane and nuclear damage (Figures 4B,C). Our findings are in line with several researchers who reported increase in electrolyte leakage in *O. sativa* (Sing and Shah, 2014), sunflower (Farid et al., 2017), and *S. sesban* (Din et al., 2020). However, the application of PGPR and earthworms reduced ROS accumulation, MDA content, and electrolyte leakage supporting the seedlings under Cr-stressed conditions. Therefore, the co-inoculation with PGPR and earthworms signifies their role in regulating cellular homeostasis and improving the membrane integrity under Cr stress.

In order to accomplish vital metabolic functions of cells, plants possessing a natural well-developed defense system known as ascorbate-glutathione (AsA-GSH) pathway, which takes place in various subcellular organelles, comprising of enzymatic and non-enzymatic antioxidants, is of prime importance that plays crucial role in combating the oxidative stress. The modulation in activities of enzymatic antioxidants might be concomitant to overcome the wrong doings of ROS and persuade tolerance to stressed plants (Hasanuzzaman et al., 2019). Our results revealed Cr stress altered the activities of antioxidant enzymes of *B. juncea* seedlings. Cr exposure enhanced the activities of SOD, POD, APX, GPOX, GR, DHAR, and GST but inhibited the activity of CAT (Table 2). The reduction in CAT activity might be due to excessive ROS accumulation, intrusion with the subunits, or altered synthesis (Bakshi et al., 2021). A similar decrease in CAT activity was observed in *Oryza sativa* (Ma et al., 2016), *B. juncea* (Mahmud et al., 2017), and *Zea mays* (Islam et al., 2016). Supplementation of earthworms and M1 and M2, alone and in amalgamation with earthworms to Cr-stressed seedlings, upregulated the activities of SOD, CAT, POD, APX, GR, DHAR, and GST except GPOX. Enhanced activity of SOD was concomitant with reduced O_2_•^–^ content as the conversion of O_2_•^–^ to H_2_O_2_ is controlled by SOD (Kaur et al., 2019). Elevated activity of POD was observed in Cr stress seedlings augmented with earthworms and PGPR, a result similar to that reported by Kaur et al. (2019) and Khanna et al. (2019). In addition, reduction in H_2_O_2_ content following the rhizospheric amendments of *B. juncea* seedlings with earthworms and PGPR alone and in combination under Cr stress was probably due to detoxification of H_2_O_2_ by CAT, POD, and APOX. Enhancement in the activities of CAT, POD, and APOX in the present study is because of the fact that these enzymes convert H_2_O_2_ to H_2_O and O_2_ and play an important role in the management of oxidative stress. Decline in GPOX activity observed upon inoculation of earthworms, M1, and M2 can be due to the involvement of CAT and APOX in scavenging H_2_O_2_, as APOX has higher affinity for H_2_O_2_ as compared to CAT and POD (Gill and Tuteja, 2010). Enhanced activities of GR and DHAR were also observed in inoculated Cr-treated seedlings. It is a potential enzyme of the ASH-GSH cycle and plays an essential role in the defense system against ROS by sustaining the reduced status of GSH. GR is also involved in the ascorbate–glutathione pathway. GR maintains the GSH/GSSH ratios by converting GSSH into GSH *via* NADPH (Noctor and Foyer, 1998). GST plays an imperative role in detoxification of heavy metals (Ghelfi et al., 2011) and catalyzes the conjugation of toxic substrates with the tripeptide glutathione (Gill et al., 2013). Similar findings were reported by Saleem et al. (2018) who found that the *Pseudomonas fluorescens* enhanced the activities of APX, SOD, CAT, and GR in sunflower grown in soils contaminated with lead. Enhanced activities of SOD, POD, and CAT were noticed in *Sesbania sesban* plant inoculated with *Bacillus xiamenensis* grown in Cr-contaminated soils (Din et al., 2020). Similarly, augmented activities of APX, CAT, POD, and SOD were observed in *Capsicum annum* seedlings inoculated with *Bradyrhizobium japonicum* under Cr^6+^ stress (Nemat et al., 2020). The expression studies of the genes for SOD, POD, CAT, APOX, GR, and DHAR in the inoculated Cr-treated seedlings are concomitant to upregulated expression of these genes at transcript levels as depicted by gene expression analysis using qRT-PCR. Inoculation of earthworms and PGPR leads to the increase in the activities of antioxidant enzymes, suggesting the affirmative attribute toward ROS detoxification and improving the ability of plants to cope up with metal stress situations.

The non-enzymatic antioxidants of AsA-GSH pathway, i.e., ascorbic acid and glutathione, are important redox buffering mediators of cell, which regularize oxidative stress by quenching ROS and conserve the redox status of the cell (Noctor et al., 2018). Moreover, ascorbic acid and glutathione are also involved in governing several developmental functions like pollen growth, cell division and differentiation, phytohormones homeostasis, etc. (Noctor et al., 2012; Potter et al., 2012). The findings of the present study showed the elevated level of ascorbic acid glutathione in Cr-treated seedlings, which is similar to the results observed in *O. sativa* (Chen et al., 2017), *B. napus* (Ulhassan et al., 2019), *Z. mays* (Adhikari et al., 2020) under Cr stress. Moreover, addition of earthworms and PGPR alone or in combination significantly upregulated the ascorbic acid and glutathione content. The results of the present study are in alliance with the findings of Gomes et al. (2013) and Islam et al. (2014), which also reported the enhancement of ascorbic acid upon strain inoculation under metal stress conditions. Fluorescent tagging of glutathione in *B. juncea* roots (Figure 4E) also depicted an increase in glutathione content upon treatment with earthworms and PGPR. Furthermore, upon addition of earthworms and M2, the most significant rise was observed. Significant augmentation in ascorbic acid and glutathione content upon inoculation of earthworms and PGPR in Cr-treated seedlings induces reduced environment in the cell, which is necessary for the survival of cell and thus assists in scavenging of ROS (Singh and Prasad, 2019). Hence, the upregulation of AsA-GSH pathway upon inoculation of earthworms and PGPR can be considered a key strategy for mitigating Cr toxicity.

## Conclusion and Future Prospects

It was concluded from the present investigation that *B. juncea* seedlings manifest reduction in biomass under Cr stress. Cr stress causes oxidative damage due to enhanced accumulation of ROS, leading to lipid peroxidation, membrane damage, and cell injury. However, the positive association of earthworms and PGPR is helpful in mitigation of Cr-induced oxidative stress in *B. juncea* seedlings. PGPR and earthworms alleviate the Cr-induced oxidative stress by modulating the activities of antioxidant enzymes and the non-enzymatic antioxidants accompanied by the decreased ROS accumulation and lipid peroxidation and improved cellular viability, thereby facilitating the seedlings to survive better under Cr stress. The present investigation provided insights into the understanding of the stress-ameliorative properties of the combined treatments of selected PGPR and earthworms by studies at three different levels. The data obtained from the bio-chemical studies, histochemical studies, and the studies of the expression of key antioxidant enzyme genes provided a holistic picture of the stress amelioration process mediated by the combined treatment of PGPR and earthworms. It will help the researchers to design-suitable strategies to enhance plant growth and productivity in the heavy-metal-affected soils. Therefore, the adoption of such techniques after optimization with the type of crop plants and the level of heavy metal stress may help in improving productivity on farm scale under the heavy-metal-affected environments. Furthermore, the identification of key players in the stress amelioration process may help in designing genetically engineered plant better suited for the high productivity under Cr stress or in designing micro- and macroorganism assisted phytoremediation of Cr polluted soils by *B. juncea*.

## Data Availability Statement

The original contributions presented in the study are included in the article/supplementary material, further inquiries can be directed to the corresponding author/s.

## Author Contributions

RB, SG, and AS designed the experiments. PS, PB, and RC performed the experiments and analyzed the data. PS and RK wrote the manuscript. RK and AS reviewed and edited the manuscript. All authors contributed to the article and approved the submitted version.

## Conflict of Interest

The authors declare that the research was conducted in the absence of any commercial or financial relationships that could be construed as a potential conflict of interest.

## Publisher’s Note

All claims expressed in this article are solely those of the authors and do not necessarily represent those of their affiliated organizations, or those of the publisher, the editors and the reviewers. Any product that may be evaluated in this article, or claim that may be made by its manufacturer, is not guaranteed or endorsed by the publisher.
